# Multimodal Physiotherapy Approach for Autism With Speech Impairment and Attention Deficit: A Case Report

**DOI:** 10.7759/cureus.50547

**Published:** 2023-12-14

**Authors:** Pradhyum D Kolhe, H V Sharath, Vaishnavi M Thakre, Prajyot Ankar

**Affiliations:** 1 Department of Pediatric Physiotherapy, Ravi Nair Physiotherapy College, Datta Meghe Institute of Higher Education and Research, Wardha, IND

**Keywords:** pediatrics rehabilitation, physiotherapy, speech impairment, attention deficit, autism disorder, children

## Abstract

Autism is a disorder distinguished by significant challenges in social interaction and communication coupled with repetitive and stereotypical patterns of behavior and activities. Deficits in social interaction and language development become apparent before age three. In children, this condition is referred to as autism spectrum disorder (ASD). Attention Deficit Hyperactivity Disorder (ADHD) is a neurodevelopmental disorder characterized by persistent patterns of inattention, hyperactivity, and impulsivity. Individuals with ADHD may struggle with sustaining attention, organizing tasks, and completing assignments. They may exhibit hyperactive behaviors such as fidgeting, difficulty staying seated, and impulsive actions like interrupting others. ADHD can significantly impact daily functioning and is often diagnosed in childhood, with symptoms potentially persisting into adulthood. The disorder has three main subtypes: predominantly inattentive, predominantly hyperactive-impulsive, and combined presentation. Treatment typically involves a combination of behavioral interventions, psychoeducation, and, in some cases, medication, aiming to provide support and strategies for individuals to manage their symptoms effectively in various aspects of life. Cognitive impairment in ASD varies, meaning it could be at the sensory perception level of cognitive processing, learning, and memory. The goal of the training intervention was to control physiological arousal, enhance awareness, keep annoyance from getting worse, and encourage self-regulation abilities. In this case report, we discuss the approach of multimodal physiotherapy for autism with speech impairment and attention deficit. Furthermore, physiotherapy needs to find a position in the new mental health care paradigm in order to contribute to mental health care.

## Introduction

Autism is a disorder distinguished by significant challenges in social interaction and communication coupled with repetitive and stereotypical patterns of behavior and activities. Deficits in social interaction and language development become apparent before the age of three. In children, this condition is referred to as autism spectrum disorder (ASD). ASD is a disorder that affects a child's neurological system, growth, and general development [1]. A child with ASD often has problems communicating; they may have trouble developing social skills. In the Diagnostic and Statistical Manual of Mental Illnesses-5 (DSM-5), ASD is a neurodevelopmental disorder characterized by persistent deficits in social communication and social interaction across multiple contexts, as well as restricted, repetitive patterns of behavior, interests, or activities. The criteria are grouped into two main domains: social communication and behavior. To be diagnosed with ASD, according to the DSM-5, an individual must demonstrate persistent difficulties in social communication and interaction, along with at least two of the four types of restricted, repetitive behaviors. The severity of ASD is specified based on the level of support required: Level 1 (requiring support), Level 2 (requiring substantial support), or Level 3 (requiring very substantial support) [2]. It is important to note that the field of psychiatry and psychology may evolve, and updates to diagnostic criteria or new editions of diagnostic manuals may occur. Therefore, it is recommended to consult the latest professional literature or experts in the field for the most up-to-date information on the diagnosis of ASD and other mental health conditions. Different approaches, especially psychosocial therapies, are used to address the core symptoms of ASD. These include specific educational programs in special education, speech, occupational, physical, and behavioral analysis, all of which have a favorable impact on the overall efficacy of treatment. Occupational therapy is frequently utilised in managing ASD, although its efficacy can be limited. Consequently, there is considerable anticipation that psychopharmacology may provide additional assistance to these individuals. The CDC states that ASD is a prevalent illness, typically first identified in early childhood [3]. The signs and symptoms of attention deficit hyperactivity disorder (ADHD), as listed in the DSM-5, include experiencing difficulties with staying focused, paying attention to details, and listening when spoken to directly. Individuals with ADHD often have trouble finishing homework and organizing projects and are reluctant to engage in demanding activities. They regularly lose critical materials needed for assignments, become easily distracted, and are restless or fidgety. Common behaviors include frequently leaving their seat in situations where remaining seated is expected, being unable to play quietly, and exhibiting restlessness as if driven by an inner motor. Additional symptoms are talking excessively, vocalizing thoughts aloud, and showing a lack of self-control or patience [4]. The level of impairment in individuals with ASD can vary, affecting perception, cognitive processing, learning, and memory [5]. Additionally, genetics plays a significant role in autism, as evidenced by the ASD occurrence rate among twins, which ranges from 36% to 95%. These genetic factors particularly influence the repetitive behavioral aspects associated with ASD [6-8]. Clinical assessments using the Indian Scale for Autism Assessment (ISAA) have yielded positive results across the board for symptoms associated with ASD [9-11]. The training intervention was designed to enhance the understanding of managing arousal, preventing annoyance from worsening, and promoting self-regulation skills [12]. It is essential to ensure that children on the autism spectrum interventions are supported by research evidence. Given the variety of accessible pharmaceutical therapies, compiling comprehensive research on interventions for children with these conditions is imperative [13]. Physiotherapists are often among the first practitioners to assess children who may be at risk for these disorders [14]. The study aims to investigate the efficacy of a multimodal physiotherapy approach in addressing the specific challenges associated with ASD, including speech impairment and attention deficit. The case report will focus on an individual with ASD who presents both speech difficulties and attention deficit symptoms. The multimodal physiotherapy intervention will incorporate a combination of physical therapy techniques, sensory integration strategies, and potentially other therapeutic modalities to enhance both motor and cognitive functions. The study seeks to assess improvements in speech abilities, attention span, and overall functional outcomes in individuals with ASD following the implementation of the multimodal physiotherapy intervention. By exploring the potential benefits of this comprehensive approach, the research aims to contribute valuable insights into the development of effective therapeutic strategies for individuals with ASD who experience speech impairments and attention deficits.

## Case presentation

Patient information

We present a case involving a three-year-old male child characterized by a spectrum of developmental anomalies. Predominant concerns encompass intellectual disability, hyperactivity, speech dysfluency, and attentional deficits. The patient was delivered at term following an uneventful gestational period of 9 months and 5 days via a lower segment cesarean section (LSCS). Postnatally, delayed initiation of the birth cry necessitated a brief admission to the neonatal intensive care unit (NICU) for observation. Challenges in breastfeeding ensued due to attentional constraints, resulting in a reliance on artificial feeding. Additionally, the patient was identified as having a calcium deficiency. At birth, he weighed 2.4 kilograms and presented with congenital anomalies, which were first observed by his mother at 2.5 years of age. Parental reports at this age indicated deficits in attention, speech fluency, social integration, and communication skills. His vocalizations remained mostly indiscernible babbling until 36 months, when he produced his inaugural linguistically coherent word. He exhibited challenges in apprehending and executing tasks and directives. Motor milestones were accomplished within normative time frames. Ambulation with support was initiated at nine months, and independent ambulation was achieved at 10 months. Equilibrium mastery was attained at 14 months. Regarding ADHD, the patient exhibited a diminished attentional span and proclivity for distractibility. Restlessness and loud vocalizations during play were evident, along with difficulty in maintaining a stationary seated position. A sensitive, temperamental disposition was noted. Modest progress was ascertained by both therapeutic interventionists and parents, with circumscribed strides in attentional endurance and communication acumen. The patient remains reliant on external feeding and continues to necessitate diaper use. Proficiency in toilet training remains unattended.

Clinical examination

The child exhibits integration with an examiner but shows less interaction with the surrounding environment. His overall activity level was average, but he exhibited a notable deficit in orientation to time, place, and person. His speech was unimpaired, and his hearing was within normal limits. However, his attention span was observed to be poor, which contributed to difficulties in maintaining focus and engagement. The anthropometric measurements are detailed in Table 1.

**Table 1:** Physical examination findings. cm: Centimeter; kg: Kilogram.

Anthropometric measures	At birth	At present
Length/Height (in cm)	10 cm	94cm
Weight (in kg)	2.40 kg	13.5 kg
Head circumference (in cm)	35 cm	46 cm
Chest circumference (in cm)	30-33 cm	49 cm

Investigation

The patient underwent a Brainstem Evoked Response Audiometry (BERA) examination, which revealed normal auditory pathway function, indicating no detectable abnormalities in the auditory system (Table 2).

**Table 2:** BERA test findings. The data has been represented as N. EP: Evoked Potentials; SPL: Sound Pressure Level; dB: Decibel; ms: Millisecond; Rec: Recorded; Cz-M: Vertex-Mastoid; R: Right; L: Left; BERA: Brainstem Evoked Response Audiometry; NA: Not Applicable.

N	Rec.sites	I Lat., ms	Ia Lat., ms	II Lat., ms	III Lat., ms	IIIa Lat., ms	IV Lat., ms	V Lat., ms	I-III Lat., ms	III-V Lat., ms	I-V Lat., ms	Stim.side	Stimulus
1	Cz-M	1.43	1.75	3.25	NA	NA	5.15	6.63	NA	NA	5.2	R	±110 dB SPL
2	Cz-M	1.53	2.85	3.35	4.6	4.85	5.25	6.68	3.08	2.08	5.15	R	±100 dB SPL
3	Cz-M	2.38	2.78	3.5	NA	4.6	5.4	NA	NA	NA	NA	R	±90 dB SPL
4	Cz-M	2.35	3.23	3.55	3.95	4.75	5.5	5.9	1.6	1.95	3.55	R	±80 dB SPL
5	Cz-M	1.73	2.45	2.8	3.75	4.03	4.3	5.83	2.03	2.08	4.1	R	±70 dB SPL
6	Cz-M	1.83	2.65	2.88	3.95	5.15	5.35	5.95	2.13	2.0	4.13	R	±60 dB SPL
7	Cz-M	2.2	NA	NA	3.75	4.35	5.68	6.63	1.55	2.88	4.43	R	±50 dB SPL
8	Cz-M	1.93	NA	NA	3.93	4.65	4.88	5.53	2.0	1.6	3.6	R	±40 dB SPL
9	Cz-M	1.35	2.83	3.3	4.4	4.68	5.25	6.78	3.05	2.38	5.43	L	±110 dB SPL
10	Cz-M	1.53	2.9	3.35	NA	NA	5.2	6.8	NA	NA	5.28	L	±100 dB SPL
11	Cz-M	1.6	2.88	3.5	4.65	4.83	5.38	NA	3.05	NA	NA	L	±90 dB SPL
12	Cz-M	2.15	3.0	3.53	4.6	4.93	5.4	5.73	2.45	1.13	3.58	L	±80 dB SPL
13	Cz-M	2.3	2.88	NA	3.9	4.8	5.33	5.78	1.6	1.88	3.48	L	±60 dB SPL
14	Cz-M	2.08	3.1	NA	NA	4.3	5.0	6.05	NA	NA	3.98	L	±40 dB SPL
15	Cz-M	1.88	3.28	3.6	NA	4.63	6.08	6.53	NA	NA	4.65	L	±50 dB SPL

Physiotherapy intervention

The patient underwent physiotherapy treatment for four weeks (Table 3). Throughout the treatment, both the patient and his family participated in counseling sessions and followed an exercise regimen.

**Table 3:** Physiotherapy management for attention deficit and speech impairment. ASD: Autism spectrum disorder; AIT: Auditory Integration Training; SIT: Sensory Integration Therapy; PECS: Picture Exchange Communication System; Min: Minute.

Sr. No.	Treatment	Description	Duration
1)	Auditory Integration Training (AIT)	AIT involves experiencing music that has been filtered and modulated, including varied volume levels and pitches.	10 min 1 session per day/week
2)	Sensory Integration Therapy (SIT)	SIT aims to enhance brain processing of information, providing a foundation for advanced skill development.	10 min 1 session per day/week
3)	Holding Therapy	Based on the theory that autism stems from insufficient bonding with the mother.	10 min 1 session per day/week
4)	Facilitated Communication	It involves a method in which a skilled facilitator provides assistance to assist a child in using an output device, like a keyboard, typewriter or similar tool to spell.	15 min 1 session per day/week
5)	Music Therapy	One of the reasons music therapy is believed to be effective for individuals with ASD is because the processes involved in improvisation can potentially aid in the enhancement of interaction and communication skills.	5 min 1 session per day/week
6)	Picture Exchange Communication System (PECS)	Its main goal is to help children who have autism learn to start conversations by giving a picture to someone they want to communicate with in order to communicate their needs.	10 min 1 session per day/week
7)	Parent-mediated Communication	In this approach, the therapist provides training to parents with the goal of enhancing their ability to understand and respond effectively to their child’s communication.	10 min 1 session per day/week

**Figure 1:**
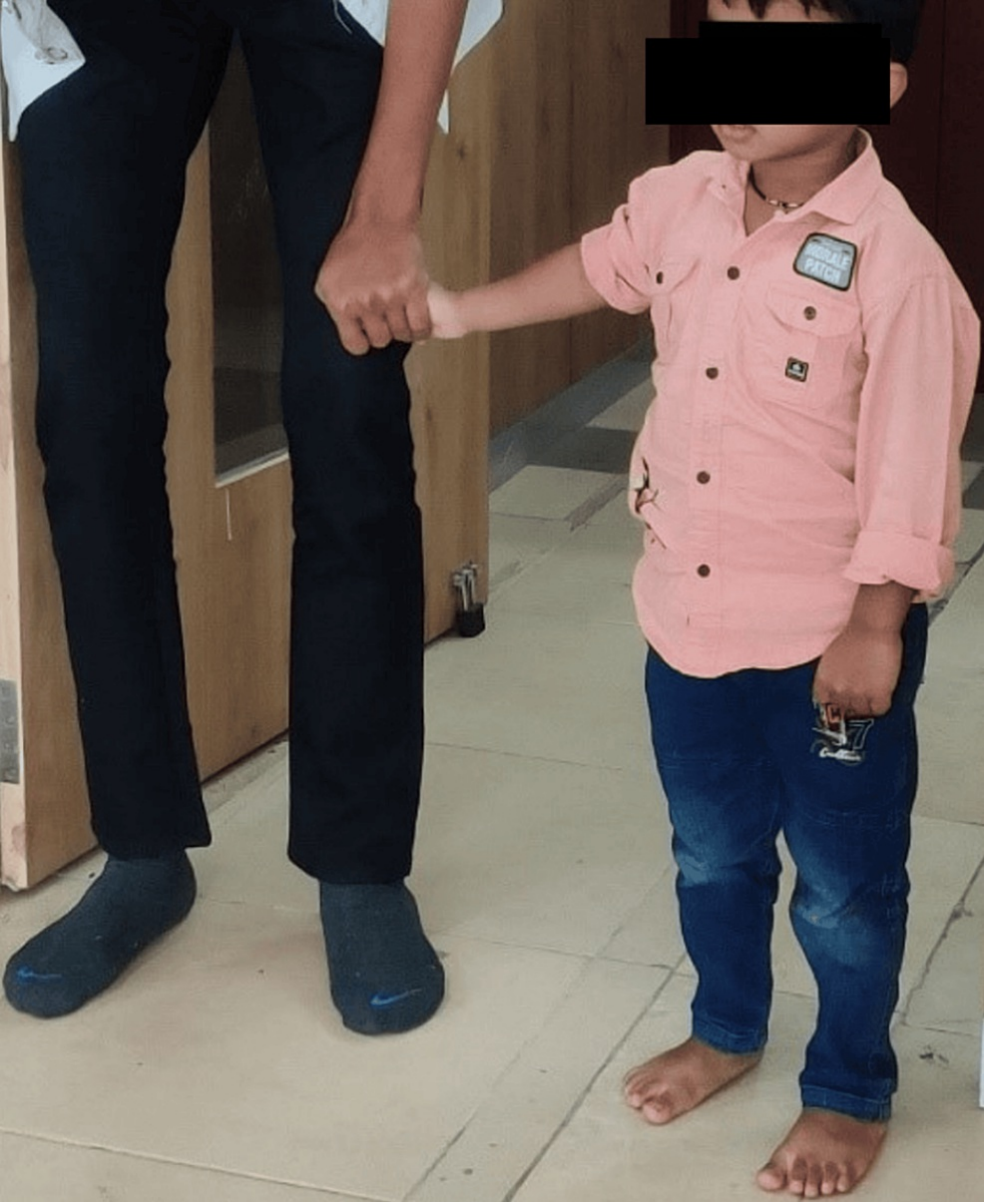
Patient undergoing holding therapy.

**Figure 2:**
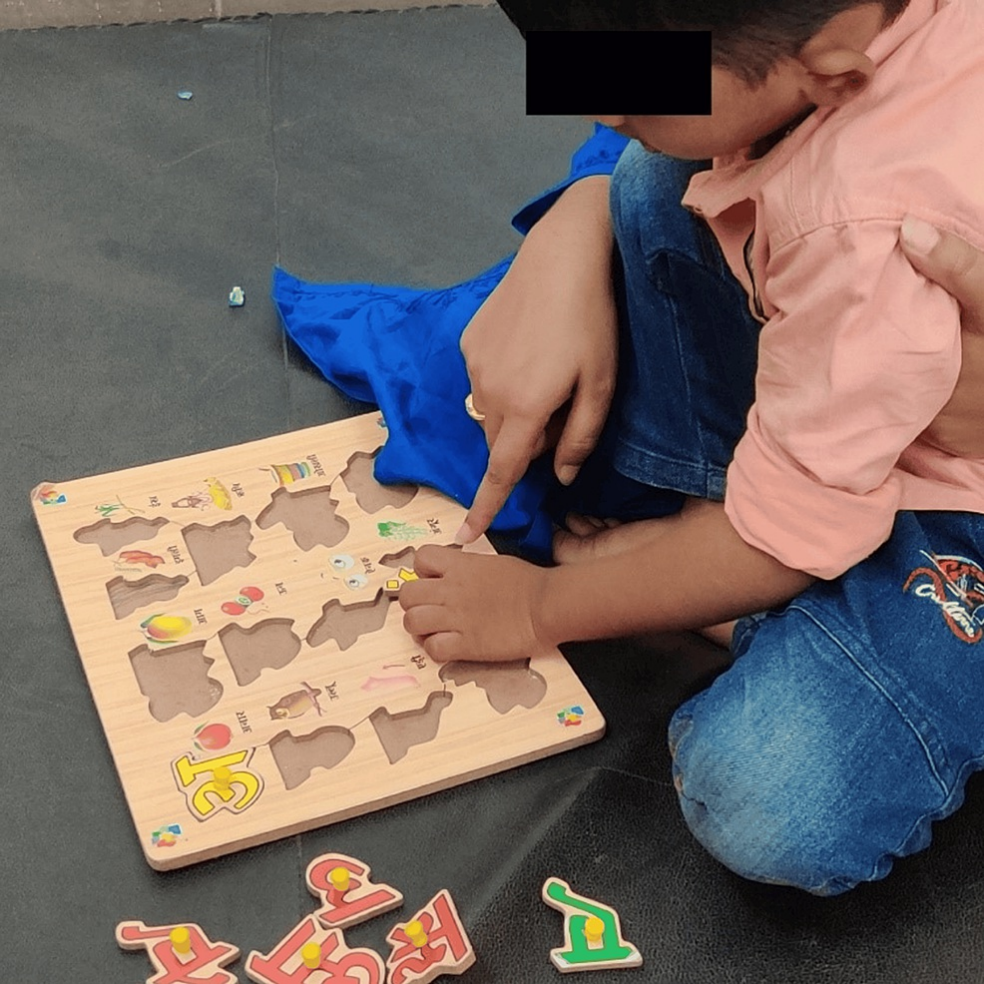
Picture Exchange Communication System (PECS).

Figure 1 shows a child undergoing holding therapy under the supervision of a therapist, and Figure 2 illustrates the Picture Exchange Communication System (PECS) therapy.

Follow-up and outcome measures

After four weeks of treatment, outcome measures were assessed. Table 4 summarizes the findings of the scales after the completion of treatment.

**Table 4:** Outcome measures. MACS: Manual ability classification system; FIM: Functional independence measures; ACS-C: Attentional control scale.

Sr. Number	Pediatrics scale	Before treatment	After Treatment
1)	MACS Scale	Level 3	Level 1
2)	WeeFIM Scale	Score 1	Score 5
3)	Conners Rating Scale	Score 4	Score 1
4)	Attentional Control Scale	Score 1	Score 4

## Discussion

This study presents intriguing findings that warrant careful consideration and interpretation. The observed improvements in speech abilities and attention deficits following the implementation of the multimodal physiotherapy intervention suggest the potential efficacy of this comprehensive approach. The integration of physical therapy techniques and sensory strategies appears to have a positive impact on both motor and cognitive functions, addressing the unique challenges faced by individuals with ASD who experience concurrent speech impairments and attention deficits. The individualized nature of the treatment plan is a notable strength, emphasizing the importance of tailoring interventions to the specific needs of individuals with ASD.

In the broader context of interventions for ASD, this study contributes to the growing body of literature exploring novel therapeutic approaches. By incorporating physical therapy and sensory strategies into a multimodal intervention, the research underscores the potential benefits of simultaneously addressing both motor and cognitive aspects. This aligns with the evolving understanding of the interconnected nature of sensory-motor functions and cognitive processes in individuals with ASD. The findings may inform clinicians and therapists working with this population, offering insights into developing more comprehensive and tailored interventions. Despite the promising results, it is essential to approach the study's implications cautiously and recognize the need for continued exploration of multimodal approaches within more extensive and diverse samples, allowing for a more robust understanding of their effectiveness across the spectrum of ASD presentations. The physiotherapy interventions presented in this protocol are a comprehensive and multidisciplinary approach to addressing attention deficit and speech impairment in a three-year-old child with developmental anomalies. Each intervention is tailored to target specific aspects of the child's condition, aiming to improve their overall communication and cognitive abilities. Auditory integration training (AIT) involves exposure to specially filtered and modulated music to enhance auditory processing, which may improve sensory integration and attention in individuals with developmental disorders [15]. Sensory integration therapy (SIT) focuses on improving the brain's processing of sensory information, particularly for children with sensory sensitivities often seen in ASD [16]. Holding therapy, based on the belief that autism is linked to a lack of maternal bonding, has its efficacy and safety debated. Facilitated communication involves a skilled facilitator assisting a child with communication challenges by using an output device for spelling words or phrases [17]. Music therapy is recognized for its potential to enhance interaction and communication skills in individuals with ASD [18]. The Picture Exchange Communication System (PECS) is an established method for children with ASD to communicate, offering a structured approach for non-verbal or minimally verbal individuals to express needs and desires using pictures or symbols [19]. Parent-mediated communication is a crucial component of the child's support system, equipping parents to effectively understand and respond to their child's communication. However, the effectiveness of each intervention can vary from one individual to another, and ongoing evaluation and research are needed to determine the long-term impact and effectiveness of these interventions. ADHD is common in children, and the implementation of therapy in mental health, complemented by interdisciplinary collaboration, is crucial [20].

The pediatric population benefits from the expertise of physiotherapists who specialize in treating children. These professionals possess a deep understanding of the interplay between early childhood development and the body's systems and functions, as well as typical child growth patterns. Physiotherapists use many advanced devices and tools, as well as generic skills, applying their additional training in development and growth to treat infants through teenagers. Physiotherapists use an extensive span of treatment approaches in the pediatrics field. In each case, careful assessment determines the treatment approach and protocol. However, it is crucial to acknowledge the study's limitations, such as the single-case design, which may restrict the generalizability of the findings. Further research with larger sample sizes and controlled designs is warranted to confirm and extend the current results. 

## Conclusions

In conclusion, this case report provides valuable insights into a holistic intervention strategy for individuals with ASD facing concurrent challenges of speech impairments and attention deficits. The observed improvements in speech abilities and attention following the multimodal physiotherapy intervention suggest the potential effectiveness of integrating physical therapy techniques and sensory strategies. The individualized nature of the treatment plan highlights the importance of tailoring interventions to the specific needs of each individual with ASD. While the findings are promising, it is crucial to recognize the study's limitations, including its single-case design, which may limit generalizability. Further research with larger and more diverse samples and controlled designs is essential to validate and extend these preliminary results. The study contributes to the broader understanding of therapeutic approaches for ASD by emphasizing the interconnected nature of sensory-motor functions and cognitive processes. The multimodal physiotherapy approach offers a nuanced perspective for clinicians and therapists, suggesting potential benefits in concurrently addressing both motor and cognitive aspects. As we move forward, ongoing research should continue to explore and refine multimodal interventions, considering their applicability across the diverse spectrum of ASD presentations and informing the development of more comprehensive and tailored therapeutic strategies for individuals with ASD experiencing speech impairments and attention deficits.
